# A consensus conference report on defining the eligibility criteria for pediatric palliative care in Italy

**DOI:** 10.1186/s13052-019-0681-3

**Published:** 2019-07-22

**Authors:** Momcilo Jankovic, Lucia De Zen, Federico Pellegatta, Pierina Lazzarin, Marina Bertolotti, Luca Manfredini, Antonino Aprea, Luigi Memo, Antonio Del Vecchio, Rino Agostiniani, Franca Benini

**Affiliations:** 1 Clinica Pediatrica, Università degli Studi Milano-Bicocca, Fondazione MBBM, ASST, Via Pergolesi 33, 20900 Monza, Italy; 2 Assistenza domiciliare e Cure palliative pediatriche AAS5 Friuli Occidentale, Pordenone, Italy; 3 Centro Regionale Veneto di Terapia del Dolore e Cure Palliative Pediatriche, Dipartimento della Salute della Donna e del bambino, Università di Padova, Padua, Italy; 4 Servizio di Psiconcologia Pediatrica, SC Oncoematologia e Centro Trapianti, AOU Città della Salute e della Scienza, Torino, Italy; 5 Centro Regionale Ligure di Terapia del Dolore e Cure Palliative Pediatriche, Istituto Giannina Gaslini, Genova, Italy; 6 Centro Specialistico di Psicologia Sanitaria e Ospedaliera, Consulente Associazione Genitin Onlus – Policlinico Gemelli, Rome, Italy; 7 UOC Pediatria, Ospedale San Martino, Belluno, Italy; 8 UOC Terapia Intensiva Neonatale – Neonatologia, Ospedale “Di Venere”, Bari, Italy; 9 UO Pediatria e Patologia Neonatale, Area Funzionale Materno Infantile, Ospedale San Jacopo, Pistoia, Italy

**Keywords:** Eligibility criteria, PPC, Consensus, Incurability

## Abstract

**Background:**

The definition of the eligibility criteria of newborn, infant, child, or adolescent patients for palliative care (PC) is complicated by the fact that these patients generally present with very specific case histories that make it inadvisable to directly adopt existing PC protocols devised for adult patients. Thus, the goal of this paper is to define a standard set of criteria for establishing pediatric palliative care (PPC) eligibility.

**Methods:**

The method adopted was that of the consensus conference. According to the guidelines issued by the Higher Institute of Health, the Board of the Italian Society for Palliative Care (i.e. steering committee) appointed a multidisciplinary group of eight health care professionals (i.e. doctors, nurses and psychologists) who worked from May 2014 to February 2016 to reach a consensus over PPC eligibility. This panel of relevant experts redacted a report summarizing all available scientific information concerning PPC, which was then submitted to the attention of a multidisciplinary jury composed of specialists and non-specialists of the field. The document thus produced was subsequently reviewed by an extended team of experts.

**Results:**

The consensus conference drafted a final document determining the guidelines for PPC eligibility of newborns, infants, children, and adolescents suffering from either oncological or non-oncological diseases.

**Conclusions:**

This report provides health care providers with practical guidelines on how to define the eligibility of pediatric patients for PPC. Given the current situation in Italy, these guidelines will be instrumental in assisting the implementation of adequate generalist and specialist PPC services as well as in helping policymakers draft and implement national legislation pertaining to PPC.

## Background

The increase in the number of children with incurable and/or severe disabilities has led to the emergence of a new category of patients characterized by special, often integrated, multi-specialist and inter-institutional care needs. For many of these young patients, the initial care offering consists of pediatric palliative care (PPC), whose primary objective is to achieve the best possible well-being and quality of life in the context of the terminal illness these young patients suffer from [1–7].

The range of illnesses making pediatric patients potentially eligible for PPC is broad and heterogeneous due to the peculiar anatomical and physiological features of these patients. Pediatric life-threatening diseases include a variety of neurological, muscular, oncological, respiratory, cardiological, metabolic, and chromosomal disorders as well as syndromes, malformations, infections, and post-anoxic conditions.

Due to disease heterogeneity, pediatric patients and their families require integrated PC programs conducted by qualified and highly specialized health care providers (e.g. play, child life, and/or child behavioral therapists) able to meet their multifaceted needs [8, 9].

In this scenario, it is becoming apparent that a definition of PPC eligibility criteria is far from being easily attained. For instance, the presence of an incurable disease cannot be regarded as a condition sine qua non for granting PPC. Instead, the decision of whether or not a pediatric patient is eligible for PPC should be made taking into account the care complexity factor, i.e., the nature and extent of the problems faced by the child and its family (e.g. clinical, psychological, social, organizational, spiritual and ethical issues) and the ensuing set of needs [10–12].

The goal of this work is to identify well-defined PPC eligibility criteria that can be easily implemented in clinical, organizational, and health care planning contexts in compliance with Law No. 38/2010 (published in the Official Journal no. 65–19 March 2010) concerning the rights of pediatric patients to pain management and PPC.

## Methods

Our approach was that of the consensus conference. It involved the consultation of a group of relevant experts who drafted a report summarizing all available scientific knowledge on PPC. This report was then submitted to a multidisciplinary jury composed of specialists and non-specialists of the field. The final document provided answers to predefined questions related to the most controversial or critical aspects of PPC eligibility and outlines recommendations for practice. The consensus conference method, unlike other approaches, allows reaching an agreement on controversial topics among renowned field experts and thought leaders in a relatively short time [13].

Specifically, a technical work-group was established within the Italian Society for Palliative Care (ISPC) consisting of 8 professionals (4 doctors, 2 nurses and 2 psychologists) with operational PPC experience in Italy. The project envisaged 4 stages of development:

1. Sharing task objectives and working methods;
2. Searching and analyzing through PRISMA (Preferred Reporting Items for Systematic reviews and Meta-Analyses) literature and documents available in the following databases:
  Primary literature databases: PubMed, Medline and Cinahil;
  Secondary Literature Banks: Trip Database and The Cochrane Library;
3. Integrating the available literature review with the clinical experience of the members of the work-group;
4. Drafting and reviewing the final document.

This task was carried out from May 2014 to February 2016 by means of meetings, conference calls and e-mail exchanges.

## Results

### Definition of incurability in pediatric oncology patients

Traditionally, a tumor is declared incurable if it is characterized by multiple therapy-resistant recurrences for which no scientific possibility of recovery is envisaged [14]. However, given the lack of shared criteria and parameters for defining the incurability of a pediatric patient, a diagnosis of incurable disease is rarely made in actual clinical practice. In this regard, the few published studies on this matter have only focused on some specific situations (e.g. the development of PPC in onco-hematology and end-of-life management in childhood cancer in Italy) [14–16]. Furthermore, in recent years new technological advances and ongoing therapeutic innovations have significantly contributed to improving recovery expectations in cases of pediatric cancer, prompting clinicians to continuously revise the definition of incurability. Finally, the process of deciding whether a patient has an incurable disease is further influenced by a host of contingent and subjective factors such as the hope for recovery on the part of the family and the difficulty in accepting failure among the medical staff despite the availability of sufficient scientific and epidemiological information in that regard. The end result of this complex decision making process is often a delay in PPC initiation, which only limits the beneficial effects of this service to terminal stages of life.

Ideally, incurability should be promptly declared as soon as the clinician is confronted with therapeutic failure and/or the impossibility of implementing a certain therapy. Such timely decision would comply with the therapeutic appropriateness criteria of the patient- and family-oriented health care model [17] and, in terms of organizational framework, with the declaration of eligibility for specialized PPC [18, 19]. In this regard, Table 1 summarizes available data and best practice comparisons concerning some specific diseases that could provide health care providers with some guidance in defining incurability in pediatric cancer patients [20].

**Table 1:** Possible situations for referring children with cancer to a PPC specialist^a^

On diagnosis	During the illness
Child with a new life-threatening illness diagnosis	Treatment-resistant disease
Extended intrinsic brainstem glioma	Progressive disease (e.g., new metastases)
Stage IV neuroblastoma	Relapsed disease after remission
Solid metastatic tumour	Resistant or recurrent disease after hematopoietic stem cell transplantation
Any other type of tumour with an event-free survival (EFS) prediction of < 40% with current therapies	Exposure to life-threatening complications (e.g., organ failure, prolonged intubation)
New diagnosis with difficult pain management or other symptoms	Development of new and significant toxicity related to treatment and/or psychosocial stress
Prolonged hospitalisation (over 3 weeks) without evidence of clinical improvement	Prolonged admission to intensive care (over 1 week) without evidence of clinical improvement

^a^minimal mod. from Kaye E, Rubenstein J, Levine D, Baker J, Dabbs D, Friebert S^20^

**Fig. 1:**
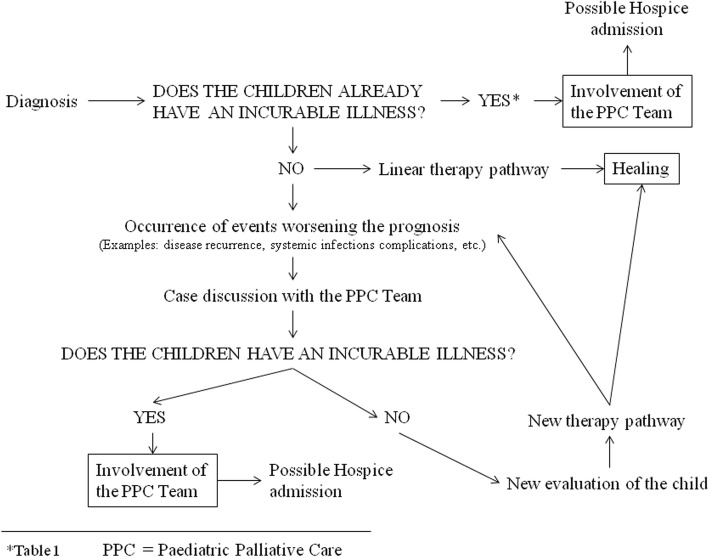
Involvement of a PPC specialist team

Given that oncology pathologies are diseases that therapies may fail to resolve, it is extremely important to address the eventuality that a diagnosis of incurability can be made at any disease stage. In this regard, team discussions attended by PPC specialists appear to be the most suitable solution for sharing information otherwise not easily attainable and for ensuring access to PPC services to all eligible pediatric patients. Below is an example flow chart for PPC specialist team involvement in infant oncology patients (Fig. 1).

### Definition of incurability in pediatric patients not affected by cancer

About 75% of children eligible for PPC suffer from non-cancer related diseases. This broad category includes children with life-limiting illnesses. For these children, the diagnosis of incurability is inherent in the underlying disorder, often represented by rare genetic diseases that usually manifest before or shortly after birth [21–24].

However, for other patients the situation is not so clear. They suffer from life-threatening disorders for which a treatment exists and may lead to a cure, but it also may prove unsuccessful. Therefore, in these cases clinicians should always be prepared to deal with a diagnosis of probable incurability by considering the need of these patients for various degrees of PPC (i.e. generalist vs. specialist PC) [25].

The last category of children is that of patients with complex and often developmental disorders, with no specific diagnosis. For these patients, it is important to consider the diagnosis of probable incurability in cases of irreversible organ failure symptoms, worsening symptoms not responding to therapy, or recrudescent events with frequent complications. Table 2 summarizes those situations requiring PPC assessment.

**Table 2:** Possible situations for referring a child with a non-cancer incurable disease to PPC

On diagnosis	During the illness
Incurable disease with terminal prognosis and risk of premature death (e.g., degenerative disease)	Illness with worsening symptoms that do not respond to therapies
Incurable disease with presence of numerous complex clinical needs	Disease with diagnosis of incurability increasing clinical/psychological/social/ethical needs
Coordination/tutoring/supervision of the welfare network to ensure assistance for the child and family	Support during the terminal phase and managing the mourning

The low frequency with which these patients are generally accepted by nursing care services can create problems for medical staff when choosing the course to be taken. Thus, teaming up with PPC specialists can strengthen the supportive effort in overcoming these difficulties.

### Defining the needs of the child and the family during the evolution of the disease

In addition to the diagnosis of incurability, the other parameters that should be taken into account are represented by all personal and social issues faced by children and their families [25, 26]. In light of this consideration, it appears that the analysis of all relevant issues would be best if carried out by a multi-disciplinary team that, by applying various skills, could assess both current and predictable problems as well as those hitherto neglected [27, 28].

## Discussion

In the literature, there is little evidence of tools—and what little is there has not been validated— suitable to evaluate the extent and therefore the weight of the assistance burden in addressing the needs and complexities of incurable children and their families. The Pediatric Palliative Screening Scale (PaPaS Scale) [29, 30] is one of these. This scale helps identify needs, and it defines a global support burden through a bio-psycho-social evaluation of the specific situation.

In this scenario, the conclusions of this consensus conference together with existing literature highlight the need for precise criteria to define eligibility of terminally ill children for PPC, which would allow clinicians to make fast and reliable diagnosis of incurable disease and PC eligibility rather than continuously delaying such decision. In this ideal situation, PPC operators could then promptly intervene in a fashion commensurate with treatment modalities (Table 3) to ensure that terminal pediatric patients receive appropriate level of care [20].

**Table 3:** ‘Green lights’ for considering a child eligible for paediatric palliative care^a^

- Child with a new life-threatening or life-limiting illness diagnosis - Difficult pain management or other symptoms - Three or more urgent hospitalisations for serious clinical crises over a period of 6 months - Prolonged hospitalisation (over 3 weeks) without evidence of clinical improvement - Prolonged admission to intensive care (over 1 week) without evidence of clinical improvement - Fitting of invasive medical devices (e.g., tracheostomy) - Child and/or family with complex psychosocial needs, limited social support or both - Child assisted by more than three specialised services, with potential interdisciplinary communication difficulties - Child with difficult and complex management of care handover between the hospital setting and the home - Child and/or family obliged to make difficult and significant decisions - Difficulties in achieving consensus between child, family and medical team on treatment and illness management goals (e.g., resuscitation actions, use of parenteral nutrition/IV hydration or continuation of chemotherapy in the terminal stages) - Child and/or family experiencing difficulty making decisions concerning resuscitation actions - Ethical debates about palliative care expressed by the child, family or medical team - Needs for continuous medical support or medical devices or frequent laboratory services by home care services if these facilities are not readily available within the primary care territorial resources - The prospect of complex outcomes in case of survival, such as a serious toxicity condition from long-term therapy - The prospect of complex needs during the mourning period	

^a^minimal mod. from Kaye E, Rubenstein J, Levine D, Baker J, Dabbs D, Friebert S^20^

## Conclusion

Although medical progress and technological advances have greatly reduced neonatal and pediatric mortality, they have simultaneously increased the survival of pediatric patients with serious and potentially lethal diseases. Despite the rise in pediatric life-limiting illnesses, there is little preparedness among health care providers on how to cope with a diagnosis of incurability requiring PPC.

This report prepared thanks to the help of the pediatric commission of the ISPC is intended as a practical guide for clarifying the designation of pediatric patients eligible for PPC and the consequent acceptance into care by dedicated specialist services.

## Data Availability

Data sharing not applicable to this article as no datasets were generated or analyzed during the current study.

## Abbreviations

ISPC: Italian society for palliative care
PaPaS Scale: Pediatric palliative screening scale
PC: Palliative care
PPC: Pediatric palliative care
